# No need for septum incision: really?

**Published:** 2020-10-08

**Authors:** Grigoris Grimbizis, Ertan Saridogan, Attilio Di Spiezio Sardo, Rudi Campo

A recent alert from a well-known scientific journal based on a published retrospective study informed the scientific community that septum incision does not improve reproductive outcome and this operation should therefore not be offered anymore as a treatment, even in symptomatic women unless as part of a clinical trial (Rikken et al., 2020) .

Indeed, such a recommendation from a high-ranked journal, officially representing a Society based on only one retrospective study was, at least, unexpected and scientifically unjustified, particularly considering that the authors of this publication are strong supporters of evidence-based medicine.

Currently, nobody (not even the authors of the above mentioned study) denies the fact that women with septate uterus have an increased risk of subfertility, pregnancy loss, preterm delivery, fetal malpresentation and intrauterine growth retardation, resulting in a higher perinatal mortality (Chan et al., 2011; Venetis et al., 2014).

Hysteroscopic septum incision represents the surgical solution for these patients; it is also important to mention that currently, the operation should be guided by data obtained by preoperative three-dimensional ultrasound imaging and should be done with mini-instruments, (scissors or bipolar needle) minimising the risk of complications (Di Spiezio Sardo et al., 2016). Thus, current surgical treatment has, of course, no relation with the historical laparotomic approaches but also differs significantly from the initial years of hysteroscopic treatment.

Current evidence based on observational studies assessing the pre- and post-operative reproductive outcome of women with septa, on non-randomized comparative studies, in which women could choose between septum resection and expectant management and prospective studies, support the notion that hysteroscopic septum incision has a beneficial effect in the achievement and evolution of pregnancy (Valle and Ekpo, 2013; Venetis et al., 2014; Mollo et al., 2009).

Based on that evidence, after many years of discussions and debates, the American Society for Reproductive Medicine (ASRM) and the National Institute for Health and Care Excellence, recommended hysteroscopic treatment of septate uterus in patients with infertility and impaired pregnancy outcomes (National Institute for Health and Care Excellence, 2015; Practice Committee of the American Society for Reproductive Medicine, 2016); a neutral position was adopted by the European Society for Human Reproduction and Embryology (ESHRE) in their recent guidelines on recurrent pregnancy losses, underlining the need for prospective data, although the detrimental effect of septate uterus on pregnancy outcome was recognized (ESHRE Guideline Group on RPL, 2018).

Thus, the question is whether a study could change the current recommended practice and why the scientists should be surprised by that published data.

Is the evidence so strong? Going through the details of the study by Rikken et al (2020), it is quite easy to recognise that the results are strongly biased; the design is retrospective in a period of almost 20 years for 21 centres (resulting in an average of 0.37 cases/centre/year). Furthermore, the diagnostic criteria for septate uterus are not defined, the diagnostic methods used have significantly different diagnostic accuracy, more infertile and less parous women were included in the treatment group, there are pregnant women (18.5% of the cases) at the time of enrolment in the expectant management group, diagnosis of the septum was made during caesarean section (~10% of the cases, all in the expectant management group), technical details of surgery in a large proportion of the treatment group is missing, etc. As a result of those biases, the study was strongly criticised (Ludwin, 2020; Saridogan et al., 2020; Alonso Pacheco et al., 2020). Thus, it should be questioned how a journal selected to promote a biased publication when it is clear that there is a need for prospective data.

Is there a need to change our practice? The published retrospective and biased data do not seem to offer an objective re-evaluation of the current available evidence as it is intergrated in the guidelines and in no way should change our practice. The symptomatic women with septa should be treated hysteroscopically.

Are there open issues? Yes there are; there is still a need to evaluate the depth of an indentation which is clinically significant and impairs women’s reproductive potential and, based on that evidence, there is a need to potentially re-evaluate the current available definitions of septate uteri (Grimbizis et al., 2014, Ludwin, 2020)

In conclusion, the published data in the aforementioned article should be viewed with great caution and the patients should not be denied the benefits of hysteroscopic septum treatment.

Grigoris Grimbizis, ESGE PresidentErtan Saridogan, ESGE Editor-in-Chief FVVOAttilio Di Spiezio Sardo, ESGE SIG Hysteroscopy CoordinatorRudi Campo, Chair, ESGE Board of Directors
